# Editorial: Dietary polysaccharides and brain health

**DOI:** 10.3389/fnut.2023.1249498

**Published:** 2023-07-25

**Authors:** Zhigang Liu, Qian Liu, Guoyuan Qi

**Affiliations:** ^1^College of Food Science and Engineering, Northwest A&F University, Xianyang, Yangling, China; ^2^College of Food Science and Technology, Northwest University, Xi'an, China; ^3^Center for Innovation in Brain Science, University of Arizona, Tucson, AZ, United States

**Keywords:** dietary polysaccharides, brain health, cognitive function, intestinal flora, age-related

Population aging is prove to be a severe social issue as the combined senior and geriatric population is projected to reach 2.1 billion by 2050. Along with the population aging intensifies, cognitive dysfunction related to aging has become a tremendous challenges. Strategies to promote healthy aging and counteract age-related brain functional degradation have become particularly important. In addition, with the rapid development of the economy and the acceleration of the pace of life, people are under increasing pressure, and the incidence of depression has soared. Cognitive dysfunction, one of the core symptoms of depression, is also a common residual symptom of depression (1, 2). Numerous studies have confirmed that nutritional strategies can reduce the risk of age-related neurodegenerative diseases and exhibit potential beneficial effects in delaying the onset of brain diseases and slowing down the progression of some conditions (3). In generally, polysaccharides are considered unable to pass through the blood-brain barrier. Nevertheless, the intestinal microbiota homeostasis regulation open new possibilities for the nutrition function of polysaccharides to brain. Numerous studies have shown that many natural polysaccharides can enrich the diversity of intestinal flora (4). However, those polysaccharides may not able to improve learning and memory function. Most of the existing studies are mainly focus on the influences of crude polysaccharide mixtures on the diversity and abundance changes of intestinal flora. The active components, structure-activity relationships, especially the molecular mechanism of dietary polysaccharides on regulating brain health are still undefined. The development of effective dietary polysaccharides nutritional interventions for maintaining brain health is becoming an emerging and challenging field.

The main goal of this Research Topic was to explore the latest outstanding discoveries pertinent to nutritional interventions of natural polysaccharides, especially in food, on cognitive dysfunction related to age/depression potential underlying mechanism. The present Research Topic includes four articles that provide in-depth insight into the effects of nutritional interventions on brain health.

A randomized, triple-blind, 69 healthy male participants, placebo-controlled trial by A randomized, triple-blind, 69 healthy male participants, placebo-controlled trial by Dalile et al. aimed to investigate the effects of extruded wheat bran (WB) on psychobiological functioning and the mediating role of short-chain fatty acids (SCFAs). The results suggested that extruded WB consumption increased serum SCFAs but did not modulate psychobiological functions in healthy men. Effective modulation of psychobiological functions may require greater increases in SCFAs than those achieved following extruded WB consumption. Rather than attempting to induce health benefits with a single fiber-rich food, combinations of different fibers, particularly highly fermentable ones, might be needed to further increase SCFA production and uptake in the systemic circulation to observe an effect on psychobiological processes.

Dietary fiber is fermented in the lower gastrointestinal tract, potentially impacting the microbial ecosystem and thus may improve elements of cognition and brain function via the gut-brain axis (5). Zhou et al. reported that L. *barbarum* berry, the total water extracts of which contain 22% polysaccharides, may be a potential antiaging natural dietary supplement especially to individuals with malnutrition or chronic diseases and a potential therapeutic agent for neurodegenerative diseases characterized by hsf-1 deficiency. Hu et al. aimed to compare the effects of soluble dietary fiber-β-glucans from mushroom, curdlan and oats bran, representing β-(1,3)/(1,6)-glucan, β-(1,3)-glucan or β-(1,3)/(1,4)-glucan, on cognition and the gut-brain axis. The correlation analysis highlights that degree of cognitive behavior improved by β-glucan supplementations was significantly associated with microglia status in the hippocampus and PFC and the number of colonic M2 macrophages. In addition, only β-glucan from oat bran altered gut microbiota and enhanced intestinal mucus.

Gut microbiota-based therapeutic strategies, such as probiotic and prebiotic preparations, may benefit mental health (6). However, commonly consumed fermented and prebiotic-containing foods have not been well-tested. A prospective cohort analysis involving 372 medical students aged 22.7 ± 1.1 years conducted by Karbownik et al. found that consumption of fermented food and food-derived prebiotics appears to be not associated with cognitive performance under psychological stress in medical students. High intake of fermented food, but not food-derived prebiotics, may be associated with severity of depressive and anxiety symptoms. The safety of fermented food in this regard therefore requires further clarification.

Overall, this Research Topic highlights recent development and innovation findings in the field of nutritional interventions of natural polysaccharides, especially in food, on cognitive dysfunction related to age/depression. The Research Topic covers the polysaccharide in food raw materials including grains, fruits and vegetables, edible fungi and the effects of these processed foods such as extrusion cooking and fermented foods on brain health. Our goal as editors was to provide a comprehensive overview of the current state of the field and identify future directions for research and development in this important area and we hope the readers will find interest in the included articles and reviews.

## Author contributions

QL wrote the article. ZL and GQ reviewed the article and provided critical feedback. All authors contributed to the article and approved the submitted version.

## References

[1] Thalamuthu A Mills NT Berger K Minnerup H Grotegerd D Dannlowski U. Genome-wide interaction study with major depression identifies novel variants associated with cognitive function. Mol Psychiat. (2022) 27:1111–9. 10.1038/s41380-021-01379-5 34782712 PMC7612684

[2] Atique-Ur-Rehman H Neill JC. Cognitive dysfunction in major depression: From assessment to novel therapies. Pharmacol Therapeut. (2019) 202:53–71. 10.1016/j.pharmthera.2019.05.013 31173838

[3] Cunnane SC Trushina E Morland C Prigione A Casadesus G Andrews ZB. Brain energy rescue: an emerging therapeutic concept for neurodegenerative disorders of ageing. Nat Rev. Drug Discov. (2020) 19:609–33. 10.1038/s41573-020-0072-x 32709961 PMC7948516

[4] Song Q Wang Y Huang L Shen M Yu Y Yu Q. Review of the relationships among polysaccharides, gut microbiota, human health. Food Res Int. (2021) 140:109858. 10.1016/j.foodres.2020.109858 33648176

[5] Frausto DM Forsyth CB Keshavarzian A Voigt RM. Dietary regulation of gut-brain axis in Alzheimer's disease: importance of microbiota metabolites. Front Neurosci. (2021) 15:736814. 10.3389/fnins.2021.736814 34867153 PMC8639879

[6] La Torre D Verbeke K Dalile B. Dietary fibre and the gut–brain axis: microbiota-dependent and independent mechanisms of action. Gut Microb. (2021) 2:3. 10.1017/gmb.2021.3 PMC11406392 39296317

